# Intrauterine food restriction impairs the lipogenesis process in the mesenteric adipocytes from low-birth-weight rats into adulthood

**DOI:** 10.3389/fendo.2023.1259854

**Published:** 2023-10-31

**Authors:** Sandra Andreotti, Ayumi Cristina Medeiros Komino, Flaviane de Fatima Silva, Ana Paula Almeida Ramos, Noemi Lourenço Gil, Gabriela Araujo Azevedo, Rogerio Antonio Laurato Sertié, Fabio Bessa Lima, Richardt Gama Landgraf, Maristella Almeida Landgraf

**Affiliations:** ^1^ Programa de Pós-Graduação em Medicina Translacional, Universidade Federal de São Paulo, São Paulo, SP, Brazil; ^2^ Department of Physiology and Biophysics, Institute of Biomedical Sciences, University of São Paulo, São Paulo, SP, Brazil; ^3^ Department of Pharmaceutical Sciences, Universidade Federal de São Paulo, São Paulo, SP, Brazil; ^4^ Department of Nutrition Sciences, University of Alabama at Birmingham, Birmingham, AL, United States; ^5^ Institute of Health Sciences, Universidade Paulista, Santos, SP, Brazil

**Keywords:** intrauterine malnutrition, white adipose tissue, lipogenesis, lipolysis, inflammation

## Abstract

**Background:**

Intrauterine food restriction (IFR) during pregnancy is associated with low birth weight (LBW) and obesity in adulthood. It is known that white adipose tissue (WAT) plays critical metabolic and endocrine functions; however, this tissue’s behavior before weight gain and obesity into adulthood is poorly studied. Thus, we evaluated the repercussions of IFR on the lipogenesis and lipolysis processes in the offspring and described the effects on WAT inflammatory cytokine production and secretion.

**Methods:**

We induced IFR by providing gestating rats with 50% of the necessary chow daily amount during all gestational periods. After birth, we monitored the offspring for 12 weeks. The capacity of isolated fat cells from mesenteric white adipose tissue (meWAT) to perform lipogenesis (^14^C-labeled glucose incorporation into lipids) and lipolysis (with or without isoproterenol) was assessed. The expression levels of genes linked to these processes were measured by real-time PCR. In parallel, Multiplex assays were conducted to analyze pro-inflammatory markers, such as IL-1, IL-6, and TNF-α, in the meWAT.

**Results:**

Twelve-week-old LBW rats presented elevated serum triacylglycerol (TAG) content and attenuated lipogenesis and lipolysis compared to control animals. Inflammatory cytokine levels were increased in the meWAT of LBW rats, evidenced by augmented secretion by adipocytes and upregulated gene and protein expression by the tissue. However, there were no significant alterations in the serum cytokines content from the LBW group. Additionally, liver weight, TAG content in the hepatocytes and serum glucocorticoid levels were increased in the LBW group.

**Conclusion:**

The results demonstrate that IFR throughout pregnancy yields LBW offspring characterized by inhibited lipogenesis and lipolysis and reduced meWAT lipid storage at 12 weeks. The increased serum TAG content may contribute to the augmented synthesis and secretion of pro-inflammatory markers detected in the LBW group.

## Highlights

* Intrauterine food restriction promotes elevated serum TAG content in 12-week-old offspring.
* Poor prenatal nutrition impairs lipid storage and attenuates lipogenesis in the mesenteric adipocytes into adulthood.
* Low birth weight 12-week-old offspring had increased IL-1, IL-6 and TNF-α gene and protein expression in the meWAT.
* Low birth weight 12-week-old offspring presented augmented liver weight and TAG content in the hepatocytes.

## 1 Introduction

The amount of adipose tissue (AT) mass in the body has been the subject of scientific attention for many years, both for its intricate relationship with obesity and, metabolic syndrome, as well as its relationship with body esthetics (1–3). The hypothesis that some diseases manifested in adulthood could have their genesis during fetal development has been proposed (4). Indeed, studies in humans and animal models have revealed strong evidence of an association between birth weight (low or high) and obesity and other diseases such as type II Diabetes Mellitus, dyslipidemia, hypertension, coronary artery disease, stroke, renal failure (glomerulosclerosis), liver failure (steatohepatitis), pulmonary abnormalities, immune dysfunction, osteoporosis, Alzheimer’s disease, depression, anxiety, bipolar disorder, schizophrenia and cancer (5–8).

A well-balanced maternal diet offers the best environment for a healthy pregnancy (9). Studies have shown that poor or restricted intake of nutrients during pregnancy is associated with abnormal fetal growth patterns, resulting in low birth weight and a higher risk for obesity and diabetes for the offspring (9, 10). This alterations into adulthood makes part of the concept known as Developmental Origins of Health and Disease (DOHaD). The first studies linking *in utero* malnutrition to adulthood obesity occurred during the “Dutch famine” between 1944 and 1945. In these studies, Ravelli et al. (11) reported that men who suffered from *in utero* malnutrition during the first half of pregnancy were more likely to become obese by the time they reached 19 years of age (11). Later, Ravelli et al. (12) demonstrated that 50-year-old women that suffered *in utero* malnutrition had higher body mass index (BMI) and waist circumference values (12).

In mammals, the AT is the primary energy depot distributed throughout the body. It is composed of several cell types, including adipocytes and cells from the stromal vascular fraction (SVF), such as pre-adipocytes, newly differentiated adipose cells, fibroblasts, leukocytes and macrophages (13, 14). The main function of all white adipocytes is to store TAG in the cytoplasm. Thus, their size is positively related to the amount of the TAG depot.

Adipocyte expansion (hypertrophy) and retraction (hypotrophy) processes are modulated by the balance between two main metabolic processes: lipogenesis and lipolysis. Lipogenesis promotes fatty acid (FA) synthesis and TAG formation. In contrast, lipolysis mobilizes FA and glycerol from TAG hydrolysis (13–15). Therefore, an imbalance in cell renewal caused by deficient or enhanced adipocyte generation (adipogenesis) or cell apoptosis may contribute to fat gain or loss. This disruption in the metabolic and/or cellular balance may or may not be accompanied by insulin resistance or other diseases (16, 17).

Despite the ubiquitous AT distribution throughout the body, the overexpansion of the visceral white AT (vWAT; in the abdominal cavity) is strongly associated with Metabolic Syndrome and other diseases. Additionally, variations in vWAT content, particularly the mesenteric white adipose tissue (meWAT) can significantly contribute to metabolic dysfunction. For example, the meWAT releases its metabolites and secretions into the superior mesenteric artery and portal vein, directly impacting the liver. Hypertrophy of this fat pad is also associated with increased inflammatory cytokine secretion, which can disrupt the WAT balance favoring lipid accumulation (18, 19).

In the present study, we investigated the consequences of a 50% reduction in the maternal diet throughout the gestational period on lipogenesis and lipolysis and sought to identify potential relationships with the inflammation process in fat pad meWAT.

## 2 Materials and methods

### 2.1 Animals

Twelve-week-old male Wistar rats from the mating of males and females supplied by the Rat Breeding Animal Farm “Zuleica Bruno Fortes”, USP, were used. The Ethics Committee on the Use of Animals (CEUA) at ICB-USP (number 112/2016) and UNIFESP (number 9816040716) approved the experimental protocol. All experiments were conducted following the ethical principles in animal experimentation adopted by the Brazilian Society of Science in Laboratory Animals/Brazilian College of Animal Experimentation (SBCAL/COBEA).

### 2.2 Mating and pregnancy

Before mating, a microscopic examination of the vaginal swab collection (VSC) was performed to detect the estrous phase. Estrus was characterized by a large number of scaly, keratinized and anucleated cells in the VSC. Two females (12-16 weeks) were placed with one male (12-16 weeks) for one night to mate. On the following morning, before removing the male, the presence of sperm after a new VSC confirmed mating and marked the first day of pregnancy. At this time, the pregnant rats were divided into two groups: 50% food restriction and *ad libitum* food intake during gestation.

### 2.3 Intrauterine food restriction protocol

The intrauterine food restriction (IFR) diet was based on a previous protocol (20–22). The amount of chow [standard commercial animal feed: 22% protein, 64% carbohydrate, 4.2% fat, 5.4% fiber, in addition to a mixture of salts and vitamins (Nuvital, Nuvital Nutrientes S/A, PR, Brazil)] provided to pregnant rats was restricted during all gestational periods by 50% and was returned to normal feeding (*ad libitum*, 100%) after the offsprings’ birth. The control group received standard commercial animal feed *ad libitum* during all gestational periods. Within 24 hours after delivery, each litter was adjusted to contain eight pups to equalize maternal care opportunities.

### 2.4 Experimental groups

All animals were kept in the Experimental Animal Facility of the Department of Physiology and Biophysics at ICB/USP. The room was maintained at 23 °C ± 2 °C with a 12-h light/12-h dark (light period starting at 8:00 am) photoperiod. The offspring were divided after the breastfeeding period into two groups: Normal Birth Weight (NBW) - cubs from *ad libitum* mothers; and Low Birth Weight (LBW) - animals from mothers who had 50% food restriction during all gestational periods. The offspring were distributed in collective cages (two animals per cage) and fed a balanced standard chow (Nuvital) and provided water *ad libitum* until 12 weeks.

### 2.5 Parameters evaluated

Twelve-week-old offspring (12-h fasted) were anesthetized with an intraperitoneal injection of 4 mg/100 g bw sodium thiopental (Tiopentax, São Paulo, SP, Brazil) and euthanized by decapitation at 8:00 am. Trunk blood samples were collected, and serum was obtained after centrifugation at 2,000 × g for 20 minutes at 4°C and stored at -80°C. Immediately after decapitation and disinfection of the animal’s abdominal wall with a 70% ethanol solution, a median laparotomy was performed to remove and weigh the heart, adrenals and WAT in the subcutaneous (scWAT), mesenteric (meWAT), epididymal (epWAT), retroperitoneal (rpWAT) regions as well as interscapular brown fat (BAT). Portions of the meWAT samples were used to verify the gene expression of lipogenic and lipolytic factors and evaluate inflammatory protein expression.

Other portions of the meWAT were used for adipocyte isolation, according to Rodbell (1964) (23), and subsequently utilized for assessing lipogenesis and lipolysis activity and cytokine secretion. The cells were photographed using an optical microscope (100×) (Moticam 1000; Motic, Richmond, BC, Canada), and the average adipocyte diameters were determined by measuring the arithmetic average of 100 cells using the Motic Images Plus 2.0 software.

The serum was used to measure the following parameters: Glucose – Liquid Glucose – (#133, Labtest, Lagoa Santa, MG, Brazil); TAG - Liquid Triglycerides (#87, Labtest); Glycerol - Free Glycerol Reagent (#F6428, Sigma-Aldrich, St. Louis, MO, USA); Non-Sterified Fatty Acids (NEFA)– NEFA-HR (2) (Wako Chemicals GmbH, Neuss, Germany); Insulin – Insulin EIA kit (#A05105, Bertin Pharma, France); Corticosterone – Milliplex Map Rat Stress Hormone Kit (#RSHMAG-69K, Magnetic Bead; Millipore, Billerica, MA, USA); Leptin - Milliplex Map Kit Cytokine/Chemokine Magnetic Bead Panel (#RECYTMAG-65, Millipore).

### 2.6 Lipogenesis activity

The adipocyte suspension (5% lipocrit) in Krebs/Ringer/Phosphate buffer, pH 7.4 with 1% bovine serum albumin and 2 mmol/L glucose, was transferred to polypropylene test tubes containing 5 μL (1850 Bq/tube) of D [U-^14^C] -Glucose and incubated at 37°C in the presence or absence of insulin (10 nmol/L). The samples were then incubated (final volume = 500 μL) for one hour in a water bath at 37°C. At the end of the incubation, 2.5 mL of Dole’s reagent (isopropanol: n-heptane: sulfuric acid, 4: 1: 0.25 vol/vol/vol) were added for lipid extraction. After vigorous stirring, 1.5 mL of n-heptane and 1.5 mL of Milli-Q water were added. The tubes were decanted, and 0.5 mL of the upper phase was collected to determine ^14^C incorporation into lipids. Radioactivity was read in a scintillation counter (1450 LSC Counter - MicrobetaTrilux, PerkinElmer, Singapore, Singapore), and the results were expressed in nmol.10^-6^ cells.h^-1^ (24).

### 2.7 Lipolysis activity

Cells (5% concentration) were incubated in Earle buffer/20 mM Hepes/1% BSA with 5 mM glucose, pH 7.4, in the presence or absence of isoproterenol (lipolytic agent). Cells were pretreated for 30 min with adenosine (0.2 μM). Then, adenosine deaminase (20 mU/mL) was added with isoproterenol (5 μM) and incubated for 30 min at 37°C. The incubation mixture (total volume of 200 μL) was centrifuged in a refrigerated centrifuge at 4° C for 5 min at 7000 rpm. Aliquots (120 μL) of infranatant were collected to determine the concentration of NEFA [Waco, NEFA-HR (2)] and Glycerol (#F6428, Sigma-Aldrich).

### 2.8 RNA extraction and quantitative real-time polymerase chain reaction

Quantitative Real-Time Polymerase Chain Reaction (qRT-PCR) was used to quantify the mRNA levels of genes involved in lipogenesis, lipolysis and inflammation. The primers used are listed in **Table 1**. Total RNA extraction was performed using TRIzol reagent (Invitrogen, Carlsbad, CA) and RNA purification kit (Ambion, Carlsbad, CA). RNA concentration and quality were confirmed by measuring the absorbance on an Epoch TM Microplate Spectrophotometer (BioTek Instruments, Winooski, VT, USA) and using the Gen5 Software. SuperScript III reverse transcriptase (catalog number: 18080-093, Invitrogen) was used for reverse transcription of 2 µg of isolated total RNA in a total reaction volume of 100 µL. Quantitative analysis of mRNA expression was performed using the StepOnePlus RT-PCR System (Applied Biosystems, Waltham, MA, USA) with TaqMan Gene Expression Assays (Applied Biosystems) at a concentration of 20 ng/µL for each sample. Gene expression analysis was performed by relative quantification using the comparative CT method (-ΔΔCT).

**Table 1:** Primers used on the meWAT qPCR.

Gene	Name Gene	Assay
Abhd5	Abhydrolase domain containing 5	Rn01446981_m1
ACACA	Acetyl-CoA carboxylase alpha	Rn00573474_m1
Acly	ATP citrate lyase	Rn00566411_m1
Adra2A	Adrenoceptor alpha 2A	Rn00562488_s1
Adra2b	Adrenoceptor alpha 2B	Rn00593312_s1
Adra2c	Adrenoceptor alpha 2C	Rn00593341_s1
Adrb1	Adrenoceptor beta 1	Rn00824536_s1
Adrb2	Adrenoceptor beta 2	Rn00560650_s1
Adrb3	Adrenoceptor beta 3	Rn00565393_m1
Agpat1	1-acylglycerol-3-phosphate O-acyltransferase 1	Rn01525981_g1
Agpat2	1-acylglycerol-3-phosphate O-acyltransferase 1	Rn01438505_m1
Aqp7	Aquaporin 7	Rn00569727_m1
Atgl	Adipose triglyceride lipase	Rn01479969_m1
B2m	Beta-2 microglobulin	Rn00560865_m1
*Cd36*	CD36 molecule (thrombospondin receptor)	Rn01442639_m1
Dgat1	Diacylglycerol O-acyltransferase 1	Rn00584870_m1
Dgat2	Diacylglycerol O-acyltransferase 2	Rn01506787_m1
*Fabp4*	Fatty acid binding protein 4	Rn00670361_m1
Fasn	Fatty acid synthase	Rn00569117_m1
*G6pd*	Glucose-6-phosphate dehydrogenase	Rn01529640_g1
Gpat	Glycerol-3-phosphate acyltransferase	Rn00568620_m1
Hsl	Hormone-sensitive lipase	Rn00689222_m1
IL1α	Interleukin 1 alpha	Rn00566700_m1
Il6	Interleukin 6	Rn01410330_m1
*Lpl*	Lipoprotein lipase	Rn00561482_m1
*Me1*	Malic enzyme 1	Rn00561502_m1
Mgl	Monoglyceride lipase	Rn00593297_m1
Pka	Protein kinase alpha	Rn01432300_g1
Plin	Perilipin 1	Rn00558672_m1
Scd1	Stearoyl-Coenzyme A desaturase 1	Rn06152614_s1
Tnfα	Tumor necrosis factor-alpha	Rn00562055_m1

Symbol, full name and test codes of the primers used.

### 2.9 Cytokine quantification

Inflammatory cytokines were determined in the tissue homogenate, culture medium and serum samples. The tissue samples were homogenized in 10 mM EDTA, 10 mM sodium orthovanadate, 2 mM PMSF and 0.01 mg/mL aprotinin twice (2655 × *g* for 20 seconds) in the Precellys instrument (Bertin Technologies, WA, USA). After extraction and separation of the cytosolic content, protein quantification was performed using the Bradford method (25).

Isolated adipocytes (5%) were incubated in 2 mL of culture medium (DMEM – D5523, Sigma-Aldrich) for 24 hours in a Lab-line incubator at 37°C, 5% CO_2_. After 24-h, 500 μL of culture medium were removed from the wells, divided into aliquots and stored at -80°C for later quantification. The Milliplex Map kit (Chemokine Magnetic Bead Panel - RECYTMAG-65, Millipore) was utilized according to the manufacturer’s instructions. The data were read with a Magpix analyzer (Luminex, Austin, TX, USA) and analyzed using the Milliplex Analyst 5.0 software (Merck Millipore, Darmstadt, Germany). Data are expressed in pg/mg of protein for homogenized tissue samples, pg/10^6^ cells/24-h for adipocytes homogenized tissue samples, pg/10^6^ cells/24-h for adipocytes in culture medium samples and pg/mL for serum samples.

### 2.10 Extraction and quantification of TAG in liver samples

Livers were removed, weighed and stored at -80°C. For TAG extraction, 100 mg of liver samples were homogenized in 2 mL of chloroform: methanol (2:1) solution (26). After TAG extraction, quantification was performed with the Liquiform Triglycerides kit (Ref. 87, Labtest) according to the manufacturer’s instructions.

### 2.11 Statistical analysis

Data were analyzed using GraphPad Prism version 8.0 for Windows (GraphPad Software, San Diego, CA, USA). All data were assessed for normality using the Shapiro-Wilk test and the “F Test” for homogeneity. Depending on the outcome, an unpaired Student’s t-test or the Mann-Whitney test was used to compare differences between groups. The fiducial limit of significance was set at 5%. In the graphs, the symbols used were: *p ≤ 0.05, **p ≤ 0.01 and ***p ≤ 0.001.

## 3 Results

### 3.1 General characteristics of animals

The general features of the experimental and control animals are presented in **Table 2**. As expected, the LBW group had less total body weight at birth than the control group (NBW animals). The 12-week-old LBW and NBW rats had similar body weights, naso-anal lengths, heart weights, adrenal weights, BAT weights, mesenteric adipocyte volumes and serum glucose, insulin and leptin levels. Notably, at 12 weeks, the weight of each fat pad (scWAT, epWAT, rpWAT and meWAT) and the percentage of these fats in relation to total body weight were reduced in the LBW group compared to the NBW group. Furthermore, the LBW group displayed elevated serum TAG and corticosterone levels compared to NBW animals.

**Table 2:** General features of animal groups.

Parameter	NBW	LBW
Birth weight (g)	6.40 ± 0.151	4.92 ± 0.214***
Body weight – 12 weeks (g)	355.7 ± 2.819	364.4 ± 6.527
Nasoanal length (cm)	24.37 ± 0.187	23.93 ± 0.208
Heart weight (g)	1.01 ± 0.033	1.09 ± 0.051
Adrenal weight (g)	0.058 ± 0.002	0.060 ± 0.002
Brown fat pad (g)	0.429 ± 0.028	0.438 ± 0.026
Adiposity (g)	18.50 ± 2.171	12.87 ± 0.828*
Adiposity/BW (%)	5.191 ± 0.600	3.497 ± 0.183*
scWAT (g)	5.209 ± 0.433	4.182 ± 0.215*
scWAT/BW (%)	1.463 ± 0.120	1.138 ± 0.043*
epWAT (g)	5.835 ± 0.759	3.990 ± 0.287*
epWAT/BW (%)	1.635 ± 0.209	1.090 ± 0.068*
rpWAT (g)	4.844 ± 0.765	2.998 ± 0.215*
rpWAT/BW (%)	1.359 ± 0.212	0.819 ± 0.054*
meWAT (g)	2.616 ± 0.262	1.811 ± 0.151*
meWAT/BW (%)	0.733 ± 0.072	0.494 ± 0.038**
Cell volume mesenteric (pL)	141.0 ± 12.80	152.3 ± 12.39
Cellularity mesenteric (×10^6^ cells)	17.37 ± 1.090	13.98 ± 0.987*
Glucose (mg/dL)	133.7 ± 9.256	118.5 ± 5.991
Insulin (IU/mL)	26.7 ± 1.417	24.93 ± 1.613
TAG (mg/dL)	33.01 ± 5.999	59.18 ± 6.105**
Leptin (pg/mL)	1202 ± 258.3	1713 ± 380.4
Corticosterone (ng/mL)	71.10 ± 20.09	333.2 ± 87.40**

Each value represents the mean ± S.E.M; for morphometric analyses - NBW (n = 13) and LBW (n = 12); for serum analyses - NBW (n = 9) and LBW (n = 12). The significance values of NBW vs. LBW were calculated using the Mann-Whitney test or unpaired Student’s t-test. *p ≤ 0.05, **p ≤ 0.01, ***p ≤ 0.001.

### 3.2 Lipogenesis

**Figure 1:**
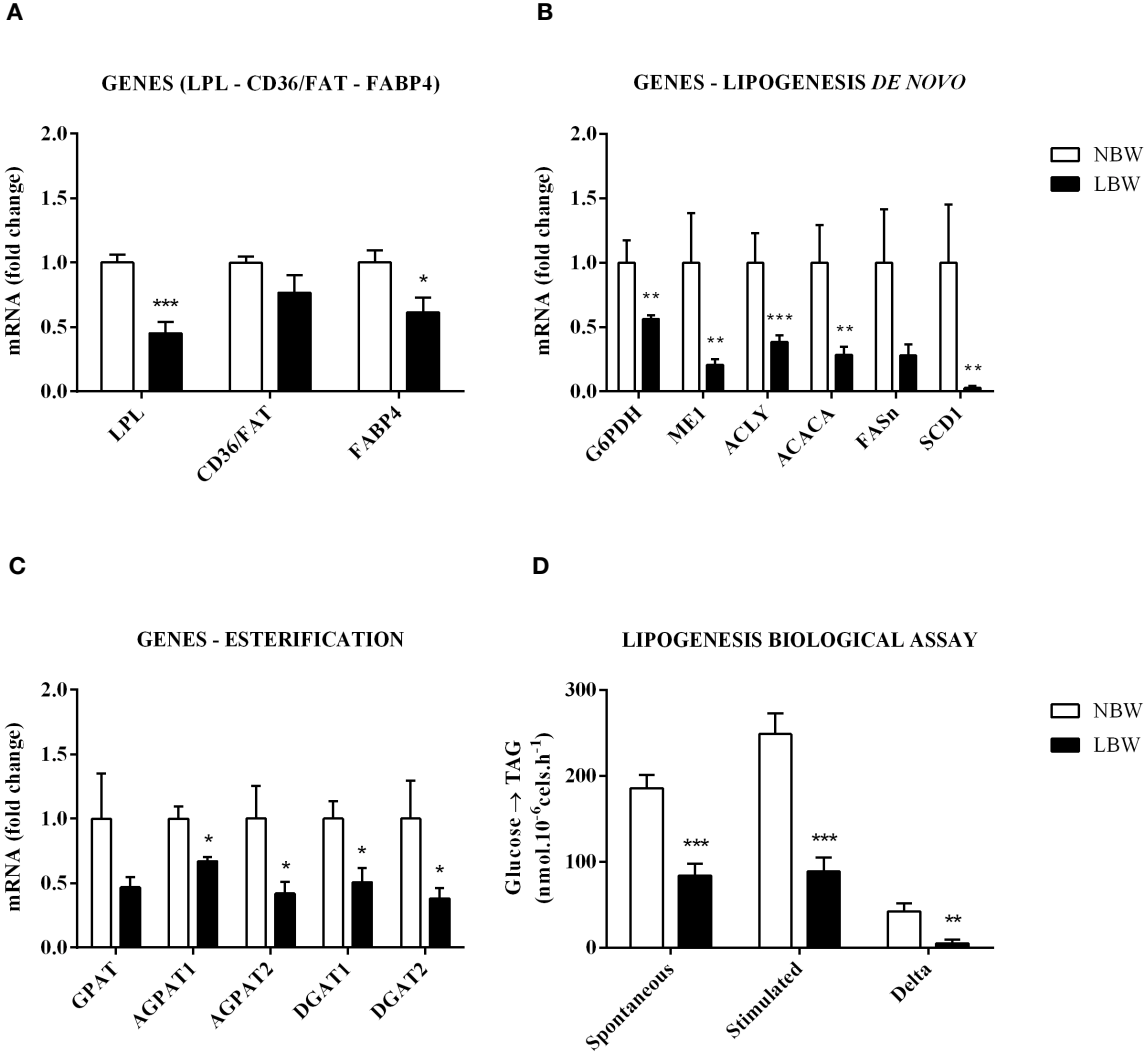
Lipogenesis in mesenteric adipocytes is reduced. **(A)** LPL, CD36/FAT and FABP4 gene expression. **(B)** Gene expression of *de novo* lipogenesis – *G6PDH*, *ME1*, *ACLY*, *ACACA*, *FASn* and *SCD1*. **(C)** Expression of esterification genes – *GPAT*, *AGPAT1*, *AGPAT2*, *DGAT1* and *DGAT2*. **(D)** Lipogenesis Assay – Basal (without insulin stimulation), Stimulated (10 nM Insulin) and Delta (Stimulated – Basal). Values are expressed as the mean ± SEM. N = 6-9 animals/group. The significance values of NBW vs. LBW were calculated using the Mann-Whitney test. *p ≤ 0.05, **p ≤ 0.01, ***p ≤ 0.001.

The lipogenesis process involves the synthesis and storage of TAG in the adipocyte’s cytoplasm. The expression of the *LPL* gene, responsible for the TAG hydrolysis in blood circulation, was reduced in LBW animals. On the other hand, there was no variation in the cluster of the *CD36/FAT* gene (responsible for NEFA uptake by the adipocytes). We also observed a significant decrease in the *FABP4* gene expression, which is responsible for the cytoplasmic NEFA transport (**Figure 1A**). Except for the *FASn* gene, which showed no significant difference between the groups, all genes related to *de novo* lipogenesis were downregulated (**Figure 1B**). These data were corroborated by functional assays using isolated mesenteric adipocytes (**Figure 1D**), which displayed decreased glucose incorporation into TAG under basal (without insulin) and stimulated (with 10 nM of insulin) conditions. As shown in **Figure 1C**, except for the *GPAT* gene (p = 0.081), all genes related to fatty acid esterification (*AGPAT1*, *AGPAT2*, *DGAT1* and *DGAT2*) were downregulated by *in utero* food restriction.

### 3.3 Lipolysis

**Figure 2:**
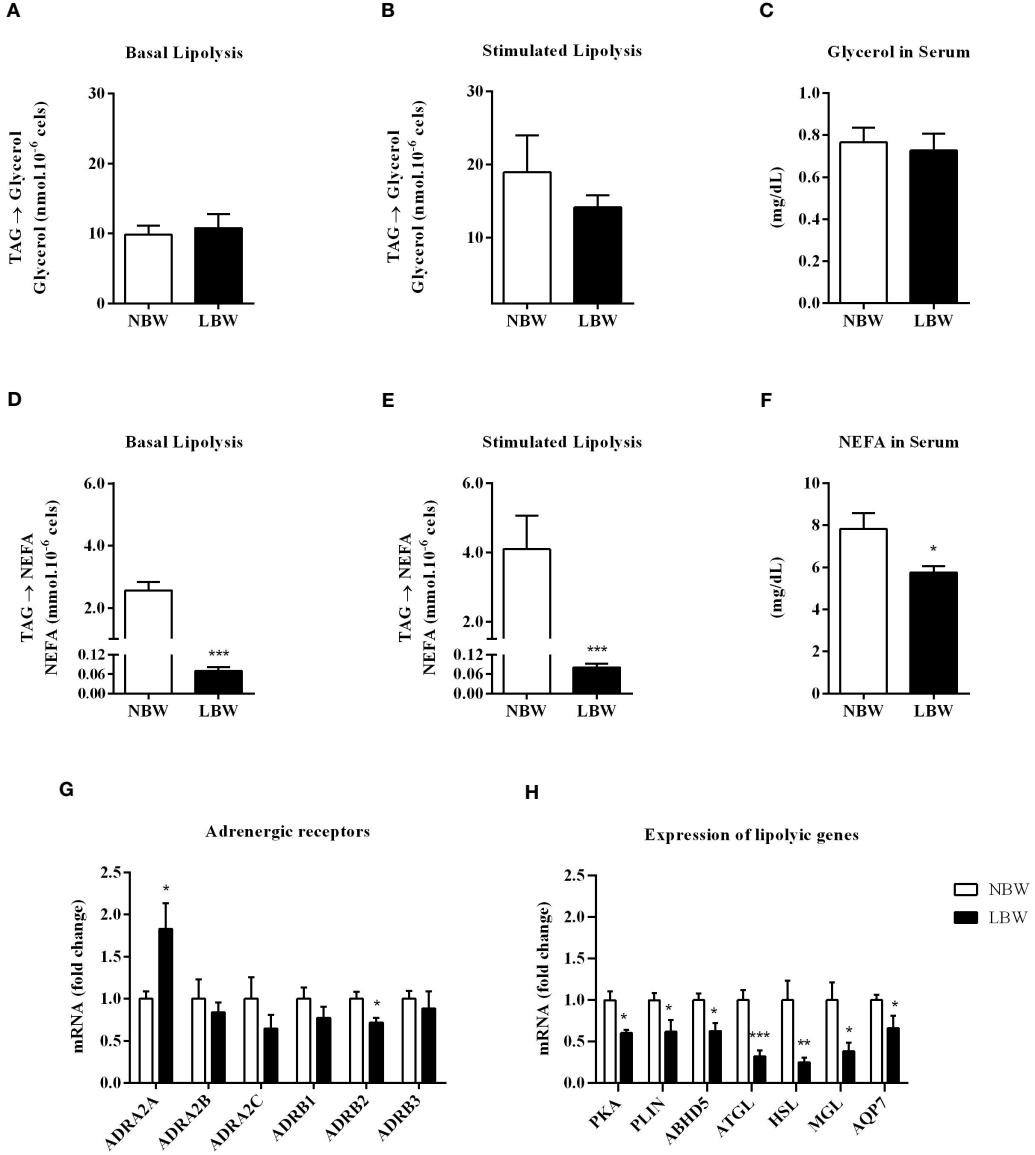
Lipolysis in meWAT. **(A)** Basal lipolysis – glycerol release. **(B)** Lipolysis stimulated (10 nM insulin) – glycerol release. **(C)** Serum glycerol content. **(D)** Basal lipolysis – NEFA release. **(E)** Stimulated lipolysis – NEFA release. **(F)** Serum NEFA content. **(G)** Adrenergic receptors gene expression – ADRA2A, ADRA2B, ADRA2C, ADRB1, ADRB2, ADRB3. **(H)** Lipolysis-related gene expression (PKA, PLIN, ABHD5, ATGL, HSL, MGL and AQP7. Values are expressed as the mean ± SEm. n = 6-10 animals/group. The significance values of NBW vs. LBW were calculated using the Mann-Whitney Test or unpaired Student’s t-test. *p ≤ 0.05, **p ≤ 0.01, ***p ≤ 0.001.

The LBW group had an impaired ability to perform lipolysis. **Figures 2A, B** indicate modifications in the lipolysis capacity (i.e., glycerol release into blood circulation). This result was verified by the quantification of serum glycerol, which was the same in both groups (**Figure 2C**). However, when assessing the functional capacity of adipocytes to carry out the lipolytic process and release NEFA, we found that spontaneous lipolysis and isoproterenol-stimulated lipolysis were reduced in the LBW group (**Figures 2D, E**). This result was further verified by the attenuated serum NEFA levels (**Figure 2F**). Additionally, in LBW animals, adrenergic receptor gene *ADRA2A* (antilipolytic gene) expression was upregulated, and *ADRB2* (pro-lipolytic gene) was downregulated (**Figure 2G**). Interestingly, the expression levels of several lipolysis-related genes, proteins, enzymes and cofactors were suppressed in the LBW group compared to the NBW group (**Figure 2H**).

### 3.4 Liver

**Figure 3:**
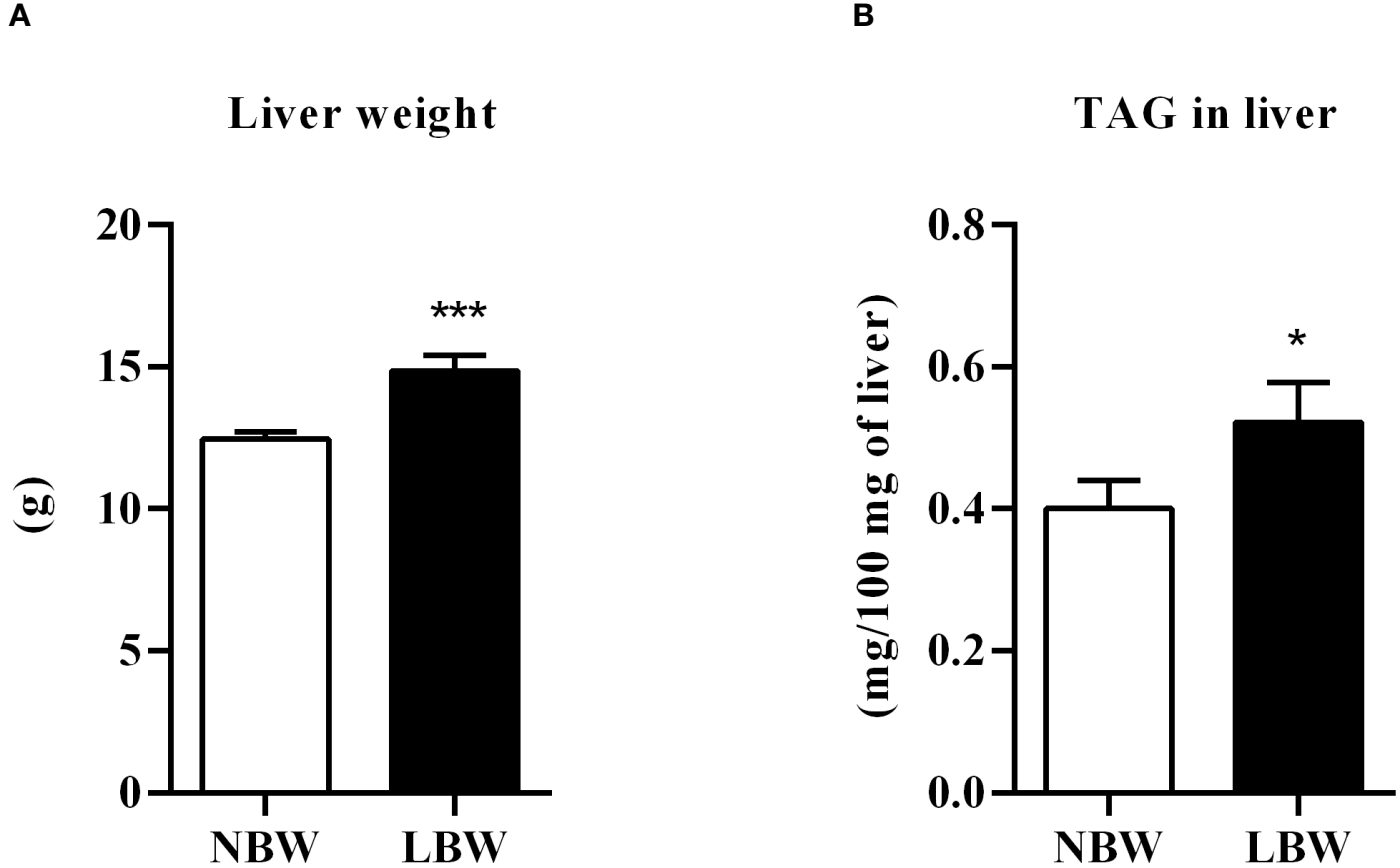
Liver. **(A)** Liver weight (g), n = 10 – 10 animals/ group; **(B)** Liver TAG content (mg/100 mg of liver), n = 14 – 10 animals/ group. Values are expressed as mean ± SEM. The significance values of NBW vs. LBW were calculated using the Mann-Whitney or unpaired Student’s t-test. *p ≤ 0.05, ***p ≤ 0.001.

As shown in **Figure 3A**, there was an increase in liver weight from LBW compared to NBW animals. This weight gain was accompanied by elevated TAG content in the liver (**Figure 3B**).

### 3.5 Secretion of pro-inflammatory markers by mesenteric adipocytes and content of these proteins in the meWAT

**Figure 4:**
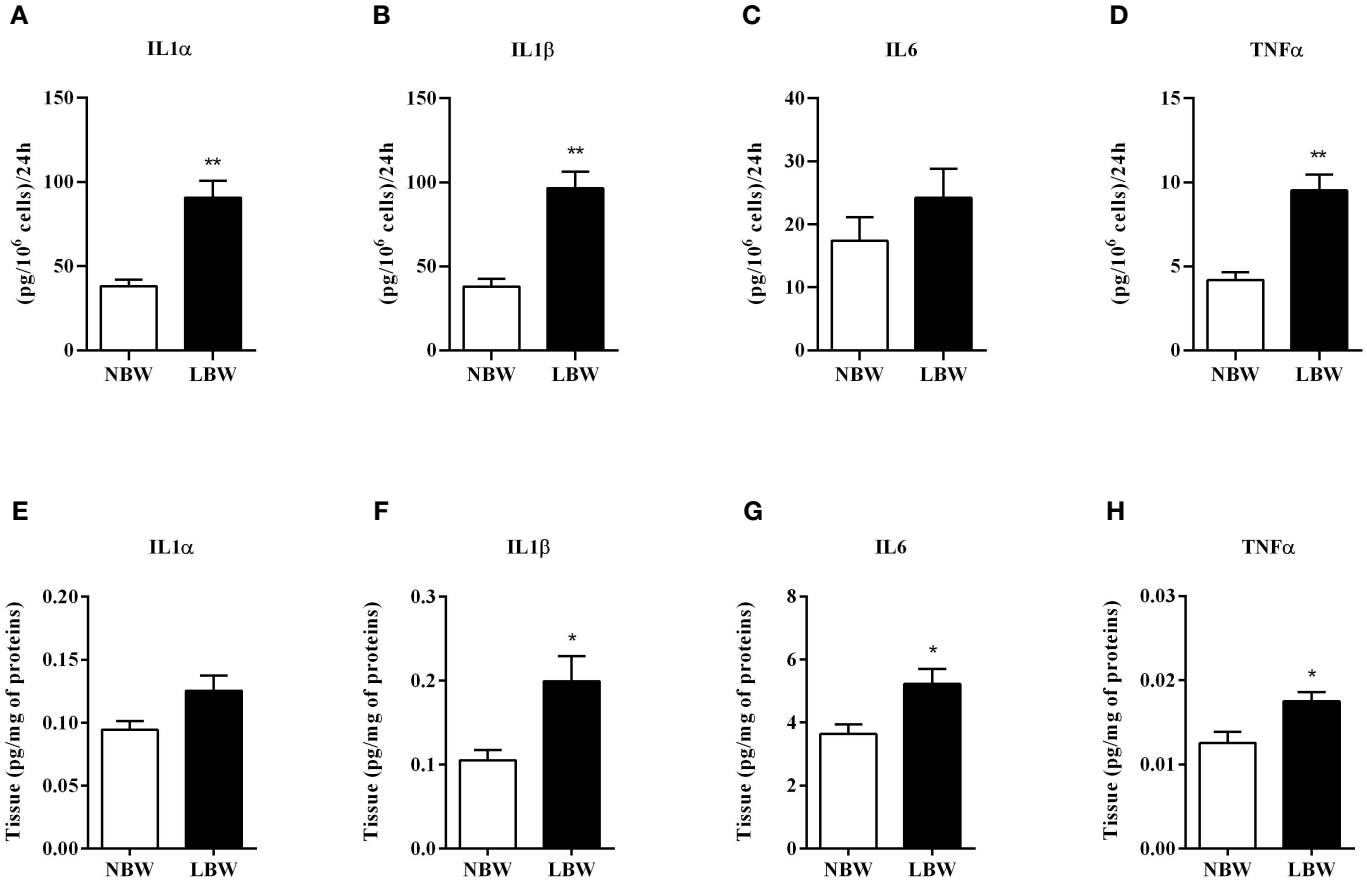
Pro-inflammatory markers secreted by adipocytes in 24 h and tissue proteins. **(A)** IL1α - secretion by adipocytes. **(B)** IL1β - secretion by adipocytes. **(C)** IL6 - secretion by adipocytes. **(D)** TNFα - secretion by adipocytes. **(E)** IL1α - proteins in tissue. **(F)** IL1β - proteins in tissue. **(G)** IL6 - proteins in tissue. **(H)** TNFα - proteins in tissue. Values are expressed as mean ± SEM. For marker secretion experiments by adipocytes, n = 4-8; for measurement of markers in tissue homogenate, n = 6-7. The significance values of NBW vs. LBW were calculated using the Mann-Whitney or unpaired Student’s t-test. *p ≤ 0.05, **p ≤ 0.01.

There was a significant elevation in the pro-inflammatory markers in LBW rats. **Figures 4A, B, D** show the interleukin secretion (i.e., IL-1α, IL-1β and TNF-α) by mesenteric adipocytes from the LBW group after 24-hour incubation. This result was further confirmed by the Milliplex experiments, which showed increased IL-1β, IL-6 and TNF-α concentration in the meWAT homogenate of LBW rats at 12 weeks (**Figures 4F–H**). There was no significant difference in the IL6 by adipocytes (**Figure 4C**) and IL1α (**Figure 4E**) in the tissue homogenate.

### 3.6 Gene expression of pro-inflammatory markers in meWAT and serum level

**Figure 5:**
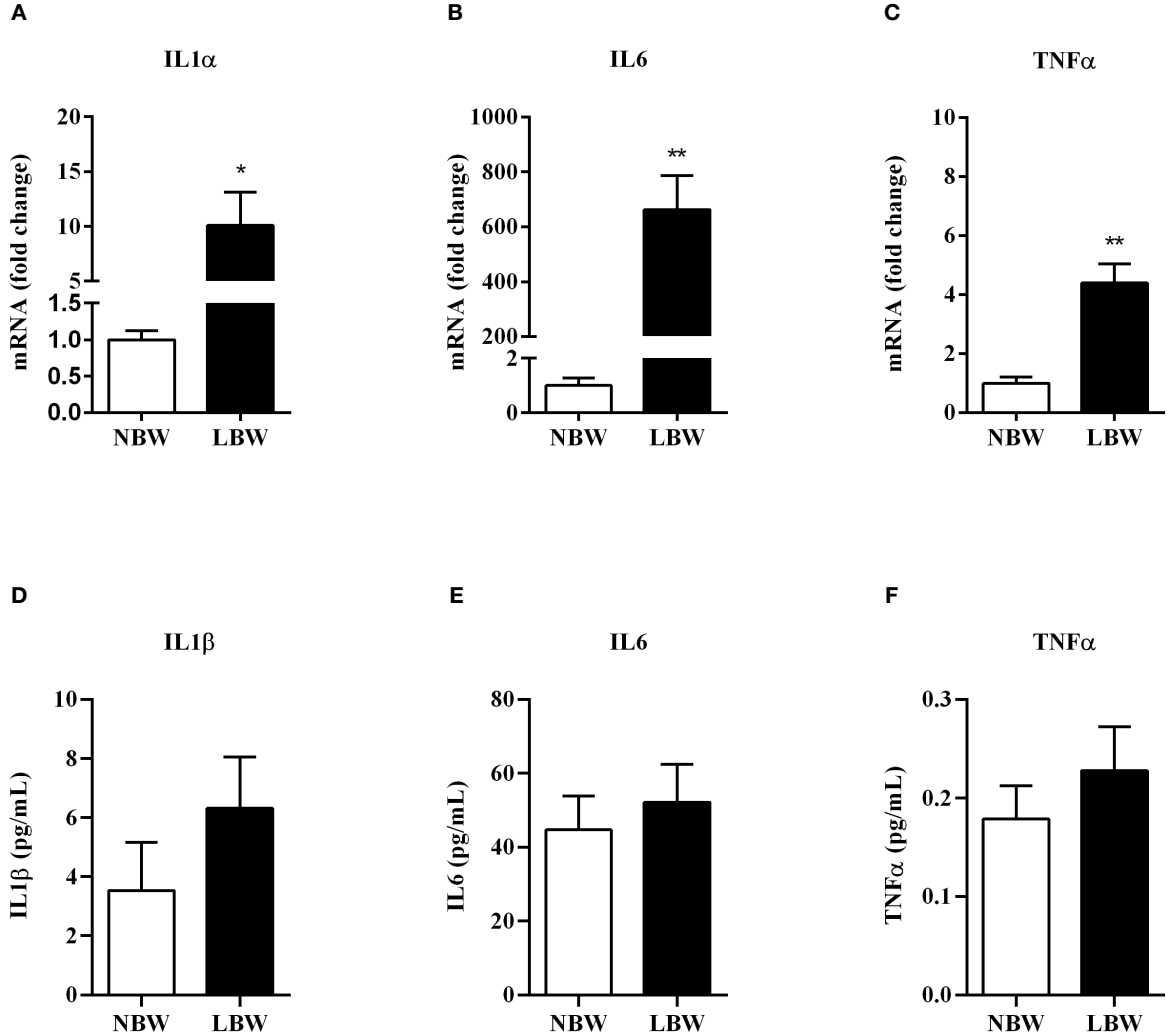
Pro-inflammatory markers gene expression and serum concentration **(A)**–*IL1α* - gene expression. **(B)**– *IL6* - gene expression. **(C)**–*TNFα* - gene expression. **(D)** IL1β – serum concentra€n. **(E)** IL6 - serum concentration. **(F)** TNFα - serum concentration. Values are expressed as mean ± SEM. For gene expression experiments, n = 6-7 animals/group; for measurements of serum interleukin concentration, n = 7-9. The significance values of NBW vs. LBW were calculated using the Mann-Whitney or unpaired Student’s t-test. *p ≤ 0.05, **p ≤ 0.01.

There was a significant increase in *IL-1α*, *IL-6* and *TNF-α* gene expression in the meWAT of LBW animals (**Figures 5A–C**). However, these alterations were not detected in the serum of the LBW group when compared to NBW animals (**Figures 5D–F**).

## 4 Discussion

There is a consensus in the literature concerning the positive correlation between *in utero* malnutrition and low birth weight. Indeed, genetic and functional modifications observed in offspring can be associated with abnormalities into adulthood, including but not limited to hypertension, excess body fat (e.g., hypertrophy and/or hyperplasia), increased circulating TAGs, insulin resistance and its associated complications, attenuated pulmonary inflammatory response and others (7, 12, 21).

The initial birth weight of the offspring from mothers who had only 50% of the total food intake during pregnancy was low. After 12 weeks, these animals had higher body mass increments than the control group, fully recovering the weight, consistent with previous studies by our group and others (20–22, 27). This phenomenon of body weight recovery in a short time is known as catch-up growth. Early postnatal catch-up growth occurring after fetal malnutrition favors the programming of obesity in adult life which in turn could be attributed to reduced fat oxidation rate and increased carbohydrate metabolism (28, 29). However, despite having the same total body weight at 12 weeks old, the WAT weight was reduced in LBW animals.

The WAT mass is closely linked with the size and/or number of adipocytes. Adipose hypertrophy and hypotrophy depend on the fine regulation between lipolysis and lipogenesis. Regarding the number of cells, which also affects the total mass of the tissue, the increase or decrease in cellularity depends on the balance between apoptosis and adipogenesis. Additionally, the increased adipose mass is related to intracellular TAG concentrations, thus contributing to the clearance of systemic TAG (13–15). In the present study, LBW animals showed a higher serum TAG serum content than NBW animals. Bieswal et al. (28) and Thompson et al. (30) also reported augmented TAG levels in 36-week-old LBW animals (29, 30).

The LPL enzyme is essential for hydrolyzing circulating TAGs and releasing NEFA and glycerol molecules that adipocytes absorb and store. Thus, the WAT plays a crucial physiological role in limiting the increment of blood fat levels by reducing NEFA release or improving the clearance of circulating TAG-containing particles, like chylomicrons or VLDLs (31–33). In LBW animals, *LPL* and *FABP4* [an intracytoplasmic transporter responsible for delivering molecules captured by adipocytes to esterification sites (15)] gene expression levels were significantly downregulated. Impaired *LPL* and *FABP4* expression could contribute to the LBW animals’ elevated serum TAG levels.

It is important to point out that detailed lipogenesis studies have yet to be performed in LBW animals, and only a few studies have reported the expression of lipogenesis-related genes and/or proteins (34, 35). Desai et al. (34) reported a significant rise in *LPL* and *FASn* gene expression followed by greater adiposity and body weight in 9-month-old animals. Similarly, Lukaszewiski et al. (35) showed upregulated *ACC* and *FASn* gene expression levels in 16-week-old LBW animals without any changes in body weight or adiposity. Herein, we found that, except for *FASn*, all lipogenesis-related genes were downregulated in LBW animals. This pathway’s suppression was confirmed by observing the reduction in the functional capacity of mesenteric adipocytes to perform *de novo* lipogenesis (DNL). The observation that adipocytes had an inadequate response when stimulated by insulin may indicate that the WAT is resistant to the hormone. Discrepancies between our data and others could be due to different experimental ADR protocols, such as the IFR during the first ten days of pregnancy or all gestational periods, the feed supplied (20%, 50% or 70% of the *ad libitum* group) to mothers during the prenatal period, and/or the age of the offspring studied (3, 16 or 32 weeks).

Another critical step in lipogenesis is esterification, a process that comprises the synthesis of TAG inside the adipocyte using three enzymes: GPAT, AGPAT and DGAT (36, 37). Except for *GPAT*, the expression of esterification-related enzymes and their isoforms were downregulated in LBW rats. This result suggests that the meWAT of LBW animals exhibits a reduced capacity to synthesize and store energy in the form of TAG, possibly contributing to the increased serum TAG levels observed in these animals.

Lipolysis is vital for energy homeostasis since it generates the NEFA necessary for several tissues through TAG hydrolysis (38). There are only a few controversial studies involving lipolysis in LBW animals. Thompson et al. (30), after IFR of 70% during all gestational periods, demonstrated that *PLIN* and *HSL* genes expression (9-month-old animals) were the same as NBW animals and, although LBW animals showed subcutaneous (SC) and retroperitoneal (RP) adipocyte hypertrophy, the functional lipolytic capacities of these cells were preserved. However, Desai et al. (34) demonstrated a rise in the gene and protein expressions of *HSL* enzyme in 9-month-old LBW animals.

The functional lipolysis results show that although the LBW group displayed adipocytes with a similar functional capacity to the NBW group, the cells performed this process less intensely. It is important to point out that NEFA may be used by the same adipocyte for the re-esterification process (NEFA to TAG), which could account for the observed NEFA content decay, as evidenced by the lipolysis assay and serum concentration. However, as previously described, the free fatty acid re-esterification process was attenuated in LBW animals. We also described that the stimulation of the lipolysis pathway by catecholamines was inhibited, and that was confirmed by the elevation in the *ADRA2A* gene expression (antilipolytic gene) and also the decrease in the *ADRB2* gene expression (pro-lipolytic beta-adrenergic receptor) in the LBW group. Finally, enzymes responsible for the TAG hydrolysis were suppressed (gene expression) in the LBW group (**Figure 2H**), corroborating our functional data (**Figures 2D, E**). According to Jaworski et al. (38), reduced lipolytic activity may contribute to the TAG accumulation in AT and consequently promote the increase in adiposity.

Thus, the drop in lipid storage measured by lipogenesis assay and the diminution in TAG hydrolysis verified through the lipolysis assay contribute to maintaining an average cell volume compatible with those observed in the NBW group. However, Fisher et al. (39) reported that the inability of visceral WAT to store excess calories in the form of TAG results in overflow and lipid ectopic accumulation in other tissues, mainly the liver, promoting local and systemic inflammation (39). This event could trigger many metabolic changes that may lead to insulin resistance, dyslipidemia and the development of metabolic syndrome. Hsiao et al. (40) also cited a study reporting that tissues such as skeletal muscle and the liver may suffer lipotoxicity when the vWAT displays the same difficulty in storing excess fat (40). Liver analyses from both experimental groups showed significant changes in LBW animals, such as increased tissue weight and enhanced ectopic fat (verified by measuring the TAG content). Therefore, the reduced ability of the meWAT to perform lipogenesis may have negatively contributed to the repercussions detected in the liver.

There is a discussion in the literature about whether obesity induces an increase in pro-inflammatory cytokines amount or if the elevation of these cytokines leads to obesity. In our experimental model, the enhanced secretion of important pro-inflammatory markers such as IL-1α, IL-1β, IL-6 and TNF-α by isolated adipocytes and their protein expression in the meWAT fat pad occurred when any change in body weight and/or increases in adiposity were observed in the offspring. Moreover, the increased synthesis and secretion of these cytokines did not result in their systemic elevation. Our findings corroborate with Egstrom et al. (2003) (41), who hypothesized that high levels of inflammatory markers occur in the early stages of the weight gain process, leading to obesity and metabolic syndrome. In this sense, the inflammation process would play a causal role in the development of the syndrome.

High levels of pro-inflammatory markers in meWAT reinforce possible variations previously analyzed. For example, TNF-α and IL-6 are cytokines expressed by different cells present in AT (i.e., adipocytes and other cell types present in the vascular stroma) that have endocrine, paracrine and autocrine effects and act in the inhibition of lipogenesis by decreasing LPL expression (42). In addition, pro-inflammatory markers promote insulin resistance by reducing GLUT4 content, insulin receptor and *IRS1* gene expression (43–45). Therefore, the augmentation of TNF-α and IL-6 cytokine content described in the mesenteric fat of LBW may have contributed to the reduced lipogenesis in these animals through LPL inhibition and local insulin resistance.

TNF-α and IL-6 are also related to non-alcoholic fatty liver disease (NAFLD), a hepatic repercussion of the Metabolic syndrome (44). Zhang et al. (46) reported that IL-1β has a direct action on the genesis of liver fibrosis (46), and Kamari et al. (47) demonstrated that the cytokines IL-1α and IL-1β play a critical role in the evolution of hepatic steatosis into steatohepatitis and hepatic fibrosis (47).

The lipolysis process can also be influenced by substances produced in the adipocyte (autocrine effect; IL-6 and TNF-α) or by other cells present in the vascular stroma, such as macrophages (paracrine effect) (48). The impact of TNF-α on lipolysis is controversial in the literature. Kettelhult and Goldberg (49) demonstrated that the TNF-α injection for five days in rats did not increase basal lipolytic activity or lipolytic activity induced by catecholamines in the WAT (49). Ruan et al. (50), after infusion of TNF-α using an osmotic pump for four days, verified a reduction in *ATGL* and *HSL* gene expression (50). However, several authors demonstrate that TNF-α (43, 51, 52), IL-6 (43, 53) and IL-1 (54) are lipolysis stimulators.

Glucocorticoids are steroid hormones secreted by the adrenal cortex under the control of the HPA axis (55). That axis is highly active in obese subjects, particularly those with central obesity (56). Our LBW animals showed a significant increase in glucocorticoid content at 12 weeks old. This data reinforces the findings of Landgraf et al. (21) and others (6, 35) who also used the IFR protocol. Additionally, hypercorticosteronemia could be related to the reduced lipolysis observed in LBW since Campbell et al. (2011) (57) demonstrated that high corticosterone concentrations could reduce or stop the lipolysis process without modifying the adipocyte viability. Also, the high corticosterone levels presented could indicate a persistent stress condition in LBW animals, possibly as a tentative way to overcome the poor metabolic response of adipocytes showed here. Glucocorticoids are strong stimulators of leptin synthesis in isolated adipocytes (58) but, in our data (at 12 weeks old) the LBW animals showed normal leptin production.

In addition to inducing pre-adipocytes differentiation into mature adipocytes and fat accumulation in different fat pads and the liver, glucocorticoids also play an essential role in growth and development (59). In the WAT vascular stroma, corticosterone leads to the differentiation and accumulation of more adipocytes, thus providing increased visceral adiposity (57). Therefore, enhanced glucocorticoid contents in these animals could account for weight gain into adulthood.

## 5 Conclusions

In conclusion, low birth weight resulting from 50% of maternal diet restriction during all gestational periods led to modifications in the metabolic processes at 12 weeks. Reduced lipogenesis may contribute to the enhanced TAG levels measured in LBW animals. Furthermore, the inability of the AT to store excess TAG may lead to ectopic fat accumulation in the liver and an increase in pro-inflammatory markers in meWAT. The rise in cytokines amount may contribute to the decrease in local insulin response. Still, the reduction in the lipolysis process observed in the LBW group, despite the increased presence of pro-inflammatory markers, may have occurred to prevent the liver from lipid overload. Furthermore, the rise in pro-inflammatory markers in the meWAT observed occurred before any genetic, functional or phenotypic clue of weight gain, demonstrating that local inflammation appears before weight gain in our experimental model. The increased circulating glucocorticoid levels found in 12-week-old LBW rats may trigger meWAT expansion and subsequent elevation in weight/adipose into adulthood.

## Data availability statement

The original contributions presented in the study are included in the article/supplementary materials. Further inquiries can be directed to the corresponding authors.

## Ethics statement

The animal study was approved by Ethics Committee on the Use of Animals (CEUA) at ICB-USP (112/2016) and UNIFESP (9816040716). The study was conducted in accordance with the local legislation and institutional requirements.

## Author contributions

SA: Conceptualization, Data curation, Formal Analysis, Investigation, Methodology, Validation, Writing – original draft, Writing – review & editing. AK: Investigation, Writing – review & editing. FF: Investigation, Writing – review & editing. AR: Investigation, Writing – review & editing. NG: Investigation, Writing – review & editing. GA: Investigation, Writing – review & editing. RS: Investigation, Writing – review & editing. FL: Funding acquisition, Resources, Writing – review & editing. RL: Conceptualization, Data curation, Formal Analysis, Funding acquisition, Methodology, Resources, Supervision, Validation, Writing – review & editing. ML: Conceptualization, Data curation, Formal Analysis, Funding acquisition, Methodology, Resources, Supervision, Validation, Writing – review & editing.
